# Health and Functioning of Community-Dwelling Older Adults in Urban and Rural Areas of Portugal—What Are the Implications for Physiotherapy Care?

**DOI:** 10.3390/ijerph22121827

**Published:** 2025-12-05

**Authors:** Magda Reis, Sara Ferreira, Monserrat Conde, Anabela Correia Martins

**Affiliations:** 1 Coimbra Health School, Polytechnic University of Coimbra, Rua 5 de Outubro, 3045-043 Coimbra, Portugal; 2 Fisioterapia, Centro Cirúrgico de Coimbra, Rua. Dr. Manuel Campos Pinheiro 51, 3045-089 Coimbra, Portugal; 3 Nuffield Department of Primary Care Health Sciences, University of Oxford, Woodstock Rd, Oxford OX2 6GG, UK; 4 H&TR-Health & Technology Research Center, Coimbra Health School, Polytechnic University of Coimbra, Rua 5 de Outubro, 3045-043 Coimbra, Portugal; 5 Center for Rehabilitation Research, Health School, Polytechnic of Porto, Rua Dr. António Bernardino de Almeida, 4200-072 Porto, Portugal; 6 SUScita—Research Group on Sustainability, Cities and Urban Intelligence, 3045-093 Coimbra, Portugal

**Keywords:** older adults, rural vs. urban, health disparities, functional capacity, fall risks

## Abstract

Background: Ageing leads to physical, cognitive, and social changes that affect people’s functioning and social participation. Health literacy, sociodemographic, and environmental factors influence health outcomes and access to care. This study aimed to characterize the health and functioning of Portuguese adults aged 65 and over, focusing on sociodemographic factors, health status, lifestyle, fall risk, functional capacity, and social participation, and on comparing rural and urban populations. Materials and Methods: An exploratory, cross-sectional study was conducted using data from older adults who completed the FallSensing screening protocol. Participants were classified by residence type (rural vs. urban), and group comparisons were made. Results: The sample (*n* = 474) was predominantly female (66.5%) with a mean age of 74.62 (±6.49) years. Rural participants were older (76.87 ± 6.89 vs. 73.50 ± 5.87) and had higher body mass index (BMI) (28.32 ± 4.31 vs. 27.51 ± 4.12), lower educational attainment—for example, 22.9% had no formal education compared to 7.0% of urban participants—and a higher prevalence of hypertension (72.6% vs. 55.4%), whereas urban participants experienced higher rates of osteoporosis (24.4% vs. 14.3%), hearing loss (41.9% vs. 26.9%), and alcohol consumption (12.7% vs. 2.3%) (*p* ≤ 0.05). Rural residents also demonstrated significantly poorer results for grip strength (21.03 ± 7.36 vs. 23.73 ± 8.61), gait speed (1.17 ± 0.44 vs. 1.45 ± 0.39), and the Timed Up and Go (TUG) test (13.4 ± 10.40 vs. 9.62 ± 4.43), as well as lower exercise self-efficacy (12.83 ± 4.97 vs. 14.28 ± 4.40) (*p* < 0.001), and more pronounced moderate-to-severe restrictions in social participation (28.0% vs. 15.7%) (*p* = 0.013). They reported greater use of assistive devices and more severe limitations in social participation. Although falls were reported more often in urban areas, rural residents experienced greater fall frequency. Conclusions: These findings suggest that rural living is associated with lower functional capacity and poorer health, underscoring the need for targeted physiotherapy and primary care strategies in rural settings.

## 1. Background

Ageing is accompanied by physical, psychological, and social changes that can compromise an individual’s health and functional capacity. Successful ageing depends on maintaining a healthy lifestyle, engaging in regular physical activity, and participating in meaningful social activities [1]. Functional capacity refers to the ability to independently perform daily activities while interacting effectively with one’s environment, especially in the home [2].

Globally, significant health disparities persist between urban and rural populations. Rural communities often experience socio-economic disadvantages, and face poorer access to healthcare, shortages of health professionals, and transportation barriers. These challenges contribute to lower levels of health literacy compared to urban inhabitants [3]. It is crucial for healthcare providers, including physiotherapists, to recognize these disparities and avoid underestimating patients’ health literacy, which significantly influences their physical activity, exercise habits, and fall risk [3,4].

Engaging in physical activity not only helps older adults improve their physical condition but also their quality of life, promoting physical, psychological, and social well-being [1,5]. Community-based approaches are particularly effective, as they enhance physical functioning and reduce social isolation [6]. Exercise also has neuroprotective benefits [7], and when individualized, is a valuable tool for both prevention and treatment of medical conditions [8].

Population ageing has become an increasingly relevant societal concern, driven by declining birth and mortality rates, reduced morbidity, and rising life expectancy. In Europe, individuals aged 65 and over represent 19.7% of the total population, a figure projected to reach 30% by 2050 [1,6]. In Portugal, 21.5% of the population is aged 65 or older, with this proportion expected to increase to 26.8% by 2030 [2].

A 2019 systematic review found that while barriers to physical activity are similar across age groups, the facilitators differ, highlighting the importance of considering age-specific factors when designing intervention strategies [9]. Additionally, both the built and natural environments have received increasing research attention due to their influence on physical activity behaviours. A 2024 systematic review reported that rural Europeans have fewer opportunities to engage in physical activity compared to their urban counterparts [10]. Similarly, a 2012 review found that Europe’s urban residents are more likely to walk or cycle for transportation, whereas rural residents primarily participate in physical activity for leisure [11].

In rural areas, the availability and accessibility of recreational facilities is positively associated with leisure-time physical activity. However, a perceived lack of safety can negatively impact walking levels [10]. Physiotherapy is vital in mitigating the consequences of ageing, particularly the risk of falls, through designing individualized therapeutic exercise plans addressing physical function [12,13]. However, sociodemographic variables such as place of residence may influence health outcomes and should therefore also be considered in care planning [9,14].

In Portugal, a study concluded that sociodemographic factors significantly impact health-related quality of life [15]. Thus, analyzing factors including place of residence is essential for developing tailored healthcare strategies that facilitate early intervention and prevent disease progression [15,16]. Despite progress in prevention and treatment, physical decline in older adults remains a challenge [6]. Assessing functional capacity and levels of dependency inform the development of self-care strategies that foster motivation, independence, and autonomy [1,17].

Nevertheless, the impact of sociodemographic characteristics on individual health remains underexplored. Considering that our environment shapes both behaviour and personality, it is essential to develop comprehensive population profiles. Such data can help primary healthcare systems to identify key intervention areas and enhance overall health outcomes.

Physiotherapy, and healthcare more broadly, can significantly benefit from studies that inform context-specific, targeted interventions. With a growing emphasis on health promotion and disease prevention, physiotherapy is playing an increasingly central role in proactive healthcare models. To effectively contribute to this paradigm shift, it is essential to identify the specific risk and protective factors linked to various health outcomes.

These factors may vary depending on sociodemographic and environmental contexts, particularly between urban and rural populations, who each face distinct challenges and opportunities in healthcare access, physical activity, and social participation. Understanding these differences is crucial for designing equitable, personalized, and geographically sensitive physiotherapy interventions supporting healthy ageing across diverse contexts.

The present study therefore aimed to comprehensively characterize the Portuguese population aged 65 and older with respect to health and functional status, focusing on sociodemographic factors, health and lifestyle indicators, fall risk, functional capacity, and social participation, while examining differences between urban and rural living environments. The findings are intended to inform healthcare planning and guide the development of tailored physiotherapy interventions addressing the specific needs of older adults in diverse residential contexts.

## 2. Materials and Methods

### 2.1. Study Design and Site

This exploratory, analytical, cross-sectional study was approved by the Ethics Committee of the Polytechnic Institute of Coimbra (registration code: 6_CEIPC_2017) and conducted in accordance with the ethical principles outlined in the Declaration of Helsinki. All the participants provided written informed consent prior to data collection.

Portuguese community-dwelling adults aged 65 years or older were recruited through parish councils and other community facilities using a convenience sampling approach. Participants were eligible if they were able to stand and walk independently, with or without walking aids, and had provided their informed consent. Individuals with severe sensory impairments (e.g., deafness or blindness) or cognitive impairment at a level that would compromise their ability to understand the questionnaires and functional tests were excluded. All individuals able to reach the screening site—regardless of educational background—were considered for participation. However, those who showed signs of cognitive impairment that interfered with test completion or raised concerns during the screening procedure were evaluated using the Portuguese version of the Six-Item Cognitive Impairment Test (6CIT) [18], and excluded if impairment was confirmed. This approach was necessary to ensure the validity and reliability of the performance-based measures and self-reported questionnaires used in FallSensing [17].

Sample size and statistical power were calculated using G*Power 3.0 (Düsseldorf, Germany). To ensure that the sample was representative of the Portuguese population aged 65 and older (approximately 2.5 million individuals), a 95% confidence level, 5% margin of error, and estimated proportion of *p* = 0.5 (maximal variability) were used. The required sample size was approximately 384 participants. A total of 474 individuals met the eligibility criteria and agreed to participate.

### 2.2. Outcome Measures

The outcome measures were part of the FallSensing screening protocol, which included both questionnaires and functional tests [17].

Questionnaires included a series of yes/no questions to characterize the participants based on sociodemographic and clinical variables, such as fall history in the previous 12 months, fear of falling, sedentary behaviour, health conditions, medication use, use of upper limbs to rise from a chair, alcohol consumption, sex, education level, and self-perceived health status.

The Self-Efficacy for Exercise Scale, a five-item questionnaire, was used to assess the participants’ confidence in performing physical activity under five emotional conditions: feeling worried or experiencing problems, feeling depressed, feeling tired, feeling tense, and being busy [19].

The Activities and Participation Profile Related to Mobility (PAPM) is an 18-item instrument, originally developed in Portugal, designed to assess the extent of difficulty individuals experience in performing daily activities in their natural environment. It covers domains such as social interactions, education, employment, financial management, and community life, all of which may influence social participation. Responses are rated on a 5-point Likert scale ranging from 0 (“no limitation or restriction”) to 4 (“complete limitation or restriction”). Not all items are necessarily applicable to every individual. A participation profile is generated based on valid responses, and the final score should be interpreted along a continuum of restriction levels. Scores from 0 to 0.19 indicate no restrictions on participation. Values between 0.20 and 0.99 reflect mild restrictions, while scores between 1.00 and 1.99 denote moderate restrictions. Scores between 2.00 and 3.87 represent severe restrictions, and scores between 3.88 and 4.00 correspond to complete restrictions on participation [20].

These two instruments were specifically chosen because they assess dimensions that are relevant in evaluating fall risk and the impact of falls on older adults. Their inclusion enables a comprehensive assessment of the behavioural and functional factors associated with falls [17], which is particularly important in understanding the potential differences between individuals living in rural versus urban settings.

Functional capacity was assessed using four standardized tests from the FallSensing protocol [17], as described in Appendix A.

### 2.3. Procedures

All the questionnaires and functional tests were administered through structured interviews conducted by two trained researchers. Both received standardized training of the FallSensing protocol [17] to ensure consistency, fidelity, and reliability. The functional tests were performed in the following order to minimize participants’ fatigue: first was Grip Strength, as it is a quick and low-effort test, and allows participants to familiarize themselves with the researcher; second was 10-Meter Walk Speed, a simple, low-fatigue functional test with minimal safety concerns; third was Sit-to-Stand, which requires greater lower limb strength, so should be performed after the less demanding tests; and fourth was Timed Up and Go, a more complex mobility test, which should be performed after participants are warmed up but before fatigue sets in.

### 2.4. Statistical Analysis

Statistical analyses were conducted using IBM SPSS™ Statistics (version 29) for Windows. Descriptive statistics, including means and standard deviations for continuous variables and frequencies and percentages for categorical variables, were computed. Missing data were excluded from the analyses. The normality of data distributions was assessed using the Kolmogorov–Smirnov test for samples with *n* > 30. Differences between urban and rural groups were analyzed using independent-samples Student’s *t*-tests or Chi-square tests, as appropriate. To control for age differences between groups, an Analysis of Covariance (ANCOVA) was conducted. A significance level of *p* ≤ 0.05 and a 95% confidence interval were applied in all statistical tests Adjustments for multiple comparisons were performed when necessary to control for Type I error.

## 3. Results

The mean age of the 474 participants included in the study was 74.62 ± 6.49 years (range: 65–95); 63.1% lived in urban areas and 36.9% resided in rural areas.

Table 1 presents the characteristics of the sample, including sociodemographic data, health conditions, lifestyle factors, alcohol consumption, and use of mobility aids. Table 2 shows fall history, and Table 3 includes social participation and functional test results.

The overall sample was predominantly female (66.5%), with no significant difference in terms of gender balance between the rural (66.3%) and urban (66.6%) participants. Rural participants were older on average (76.9 ± 6.9 years) than urban participants (73.5 ± 5.9 years).

Regarding educational attainment, nearly half of the participants (47.0%) had completed only primary school, while 7.8% had completed secondary education and 6.9% had higher education; 12.9% were illiterate. Stratification by residence revealed notable differences: in rural areas, the majority (57.7%) had completed only primary school, and illiteracy was higher (22.9%), whereas urban participants had a more diversified educational profile, with lower illiteracy (7.0%).

A substantial proportion of participants lived alone (43.5%), with a slightly higher prevalence in rural areas (46.5%) than urban areas (38.3%).

Hypertension was the most commonly reported health condition (61.7%), particularly among rural participants (72.6% vs. 55.4% in urban areas). Osteoporosis and hearing problems were more prevalent in urban areas (24.4% and 41.9%, respectively), whereas visual impairment was more frequent in rural participants (68.0% vs. 54.8%). Other conditions, including hypercholesterolemia, osteoarthritis, urinary incontinence, diabetes, myocardial infarction, stroke, and Parkinson’s disease, did not differ significantly between areas.

Self-rated health was most commonly reported as fair or good, with only urban participants reporting excellent health; overall, no significant differences were found between rural and urban participants.

Benzodiazepine use was similar across areas (29%), while polypharmacy (the use of more than four medications) was more common in rural areas (71.4% vs. 56.5%). Mobility assistive device use was higher in rural areas (20.0% vs. 9.4%), with crutches and canes being the most frequently used devices.

Over half of the participants were classified as sedentary (52.7%), and 43.2% used their upper limbs to rise from a chair, an indicator of lower-limb functional decline, with no differences between rural and urban areas.

Body mass index was slightly higher in rural participants (28.3 ± 4.3 kg/m^2^ vs. 27.5 ± 4.1 kg/m^2^), and alcohol consumption was more prevalent in urban areas (12.7% vs. 2.3%).

Overall, 185 participants (39.0%) reported having had at least one fall in the previous 12 months, with a slightly higher proportion in urban areas (40.5%) compared to rural areas (36.6%), although this difference was not statistically significant. Fear of falling was reported by 261 participants (55.1%), with similar prevalence in rural (60.6%) and urban (51.8%) groups.

Regarding the location of falls, the majority occurred outdoors (65.8%), with comparable distributions between rural and urban participants. The participants identified several causes for their falls, with stumbling most frequently reported (42.4%), followed by slipping (23.9%), loss of lower-limb strength (7.1%), dizziness (6.5%), and loss of consciousness (1.6%). A small proportion could not identify a specific cause, or cited other factors. Patterns were similar across rural and urban areas, with stumbling and slipping predominating in both, and loss of consciousness only reported by urban participants.

Following a fall, 34.1% of participants sought medical attention, most of whom visited a hospital, and a subset required hospitalization. Rural and urban participants showed similar patterns in seeking care and hospital visits.

As a consequence of falling, 23.4% of participants reported limitations in activities or restrictions in participation, either in the short or long term, with similar rates between rural and urban areas.

Self-efficacy for exercise also differed between areas, with rural participants reporting lower scores (12.83 ± 4.97) than urban participants (14.28 ± 4.40), confirming a significant difference.

According to the PAPM results, half of the participants reported no restrictions in social participation, while smaller proportions reported mild, moderate, or severe restrictions. Rural participants tended to report more moderate and severe restrictions compared to urban participants, reflecting a significant difference between the groups.

Functional tests revealed notable disparities between rural and urban participants. The overall mean TUG time was 10.99 ± 7.40 s, with rural participants performing slower than urban participants. Mean grip strength was lower in rural areas (21.03 ± 7.36 kg) compared to urban areas (23.73 ± 8.61 kg). Walking speed averaged 1.35 ± 0.43 m/s, with rural participants walking more slowly (1.17 ± 0.44 m/s) than urban participants (1.45 ± 0.39 m/s). In the 30-s sit-to-stand test, rural participants performed worse than urban participants. All functional differences between the groups were statistically significant.

Analyses of covariance (ANCOVA) were conducted to examine the effects of age and place of residence (rural vs. urban) on health conditions, lifestyle behaviours, mobility aids, social participation, functional capacity and self-efficacy for exercise outcomes. Age was included as a covariate in all models (see Appendix B).

## 4. Discussion

Portugal is experiencing pronounced demographic ageing, which is creating significant challenges for healthcare planning and resource allocation [1,3]. This cross-sectional study provides an updated snapshot of the health, functional status, and social participation of older adults living in rural and urban settings, offering baseline evidence to guide ageing-in-place strategies, including physiotherapy care.

Our findings highlight substantial rural–urban disparities. Rural participants were on average older, had higher BMI, lower educational attainment, higher rates of illiteracy, and more frequent polypharmacy. They also had a higher prevalence of hypertension, poor vision, and mobility aid use. In contrast, osteoporosis, hearing problems, and alcohol consumption were more common among urban residents. Across both groups, more than half of the participants reported hypercholesterolemia, osteoarthritis, sedentary behaviour, and fair or poor self-rated health. Diabetes and urinary incontinence affected roughly one third of the sample.

Although falls (and recurrent falls) were more frequent in urban areas, rural residents had poorer physical performance across all functional tests, lower exercise self-efficacy, and a higher proportion of moderate-to-severe social participation restrictions. Fear of falling affected over half of the participants. Slipping or tripping accounted for most falls.

The high burden of multimorbidity observed here aligns with previous population-level studies in Portugal, which have reported multimorbidity affecting nearly half of adults aged 50 or older [21]. Differences in disease patterns, functional performance, and lifestyle behaviours across rural and urban areas echo findings from other European and international studies, where healthcare access, socioeconomic factors, and environmental conditions shape ageing trajectories [16,22,23,24,25,26].

Earlier research has similarly reported the detrimental effect of fear of falling on autonomy, functional capacity, social participation, and quality of life, reinforcing its cyclical relationship with fall risk [27,28,29]. Consistent with previous studies, we found strong associations between functional decline and rural residence, likely reflecting both age differences and environmental constraints [22,30,31].

Our findings also align with evidence showing that outdoor falls represent a substantial proportion of medically attended fall incidents, at rates sometimes exceeding indoor falls [32]. We identified twice as many outdoor as indoor falls, consistent with prior literature suggesting the importance of environmental hazards in fall events [24,33,34,35], particularly in urban settings.

As a recent systematic review concluded, micro-walkability (for example, the quality of pavements or road surfaces) remains the most frequently studied external environmental factor. However, macro-accessibility (e.g., the use of spaces) from the perspectives of crime and safety, socio-economic characteristics, green spaces, and terrain has been less studied and has yielded contradictory results [36,37,38,39]. As well as undoubtedly being useful in identifying areas where intervention is needed, we argue that this macro-scale can be specifically useful in classifying existing or proposed neighbourhoods in terms of their walkability.

Furthermore, social isolation in older adults, which is estimated to affect a quarter of the global older population [40], was also reflected in our sample, with more than 40% of participants living alone. Prior studies corroborate the link between living alone, limited social contact [41,42], frailty, comorbidities, and fear of falling [43].

At this point, it is important to recall the World Health Organization’s definition of healthy ageing as the process of developing and maintaining the functional ability that enables well-being in older age, alongside the four priority action areas identified in the Decade of Healthy Ageing [37]. Our findings underscore the urgent need to transform societal attitudes towards ageing, to foster environments that support older adults’ abilities, to deliver person-centred and integrated primary healthcare which is responsive to their needs, and to ensure access to long-term care for those requiring it. Physiotherapy services must also address rural–urban disparities in efforts to enhance functional ability, improve quality of life, and promote healthy ageing in Portuguese older adults [22,23].

Physiotherapists should be equipped to conduct context-sensitive assessments that consider the social determinants of health influencing older adults’ profiles [44], and to implement interventions aimed at enhancing functional independence, supporting autonomous living, and reducing falls and fear of falling. Such interventions include home modifications, patient education and coaching, and structured exercise programmes [45]. For rural populations, physiotherapy must be adapted to overcome barriers such as transportation difficulties, limited healthcare access, and lower health literacy [41], through culturally tailored, home-based, hybrid, and telehealth strategies. Programmes should be designed for low-resource settings, drawing on community assets to ensure feasibility and sustainability [46]. Finally, physiotherapy curricula for geriatric care should embed these competencies at both postgraduate and continuing professional development levels.

Given the striking disparities between rural and urban areas, our findings provide practical guidance for physiotherapeutic assessment and intervention planning, highlighting the need for specific assessments that consider the physical, social, and attitudinal context, and use targeted fall prevention strategies and adaptable service delivery models—including home-based and telehealth approaches—to ensure equitable and effective care for older adults, particularly in rural settings marked by scarce public health services resources, including many areas without a physiotherapist—and even when one is present, they may be unable to provide timely responses that ensure sustainable outcomes.

This study has limitations. The cross-sectional design precludes causal interpretation and limits temporal understanding of health and functional changes. Although this design does not allow examination of long-term trajectories, it provides valuable data for immediate care planning.

While a power calculation supported our sample size, Portugal exhibits marked regional socioeconomic and cultural heterogeneity, which may influence both rural and urban ageing experiences. Variations in income, geography, and healthcare access contribute to unequal health outcomes and may limit the generalizability of our findings [47,48]. Excluding individuals with cognitive impairment may have introduced selection bias, potentially overestimating the functional performance of the sample. In addition, recruitment bias may have occurred, as people who agreed to participate could differ systematically from those who did not, potentially affecting the observed outcomes. Age differences between rural and urban participants (of approximately 3.5 years) may have influenced functional outcomes due to age-related decline [49,50,51]. However, the findings suggest that both age and place of residence contribute to variations in physical function, health, and psychosocial outcomes, with age predominantly influencing functional decline and certain health measures, while the rural-urban context appears to play a more prominent role in psychosocial factors, medication use, and some health conditions.

Future studies should adopt longitudinal designs to track changes in health, functional ability, and social participation over time, and to evaluate how community-based physiotherapy interventions and environmental modifications can mitigate disparities.

## 5. Conclusions

Our findings suggest that rural living is associated with poorer outcomes among Portuguese older adults in most health and functioning domains, reinforcing the need to implement comprehensive physiotherapy assessments that consider the social determinants of health.

To promote equality between rural and urban populations and reduce differences between them, accessible and community-focused programs are essential. Thus, we emphasize the high relevance of physiotherapy, mainly exercise by prescription, in primary and community settings to promote functional capacity and healthy lifestyles. Although age influences social participation and the use of mobility assistive devices, many other characteristics of older adults, which may also be age-dependent, are equally affected by their place of residence. To the best of our knowledge, this study is the first to provide relevant data for consideration by physiotherapists in Portugal, to develop sustainable strategies for early intervention on aspects of the ageing process that are influenced by whether an individual resides in a rural or urban area.

Future research should disentangle age effects through stratified analyses and explore additional health determinants, including socioeconomic and psychosocial factors. This information will support the design of targeted, person-centred physiotherapy interventions tailored to the specific needs of older adults living in different environments.

## Figures and Tables

**Table 1:** Sociodemographic characteristics, health conditions, lifestyle behaviours, and mobility aids, overall and stratified by living environment (rural vs. urban).

Characteristics	Overall (*n* = 474)	Rural (*n* = 175)	Urban (*n* = 299)	*p*
Age (years), mean (SD)	74.6 (6.5)	76.9 (6.9)	73.3 (5.9)	<0.001
Female, *n* (%)	315 (66.5%)	116 (66.3%)	199 (66.6%)	0.952
Education, *n* (%):				<0.001
No education	61 (12.9%)	40 (22.9%)	21 (7.0%)	
Up to 4 years	223 (47.0%)	101 (57.7%)	122 (40.8%)	
5 to 9 years	150 (25.3%)	17 (9.7%)	103 (34.4%)	
Completed high school	37 (7.8%)	8 (4.6%)	29 (9.7%)	
University	33 (6.9%)	9 (5.2%)	24 (8.0%)	
Living alone (Yes), *n* (%)	206 (43.5%)	67 (38.3%)	139 (46.5%)	0.082
Heart attack (Yes), *n* (%)	23 (4.9%)	8 (4.6%)	15 (5.0%)	0.828
Stroke (Yes), *n* (%)	31 (6.5%)	14 (8.0%)	17 (5.7%)	0.325
Diabetes (Yes), *n* (%)	144 (30.4%)	55 (31.4%)	89 (29.8%)	0.704
Hypertension (Yes), *n* (%) (*n* = 473)	292 (61.7%)	127 (72.6%)	165 (55.4%)	<0.001
High cholesterol (Yes), *n* (%) (*n* = 473)	265 (56.0%)	97 (55.4%)	168 (56.4%)	0.841
Osteoarthritis (Yes), *n* (%)	239 (50.4%)	89 (50.9%)	150 (50.2%)	0.885
Osteoporosis (Yes), *n* (%)	98 (20.7%)	25 (14.3%)	73 (24.4%)	0.009
Urinary incontinence (Yes), *n* (%)	179 (37.8%)	61 (34.9%)	118 (39.5%)	0.318
Hearing problems (Yes), *n* (%) (*n* = 473)	172 (36.4%)	47 (26.9%)	125 (41.9%)	<0.001
Poor vision (Yes), *n* (%)	283 (59.7%)	119 (68.0%)	164 (54.8%)	0.005
Self-perceived health, *n* (%) (*n* = 389):				0.303
Poor	71 (18.3%)	20 (18.0%)	51 (18.3%)	
Fair	170 (43.7%)	53 (47.7%)	117 (42.1%)	
Good	109 (28.0%)	31 (27.9%)	78 (28.1%)	
Very good	29 (7.5%)	7 (6.3%)	22 (7.9%)	
Excellent	10 (2.6%)	0 (0%)	10 (3.6%)	
Benzodiazepines (Yes), *n* (%) (*n* = 419)	123 (29.4%)	49 (29.9%)	74 (29.0%)	0.851
Total medication, mean (SD) (*n* = 351)	5.37 (2.89)	5.58 (2.95)	5.16 (2.82)	0.192
More than 4 medicines (Yes), *n* (%)	294 (62.0%)	125 (71.4%)	169 (56.5%)	0.001
Mobility assistive device (Yes), *n* (%)	63 (13.3%)	35 (20.0%)	28 (9.4%)	<0.001
Type of assistive device *n* (%) (*n* = 63):				0.710
Walking stick/cane	26 (41.9%)	15 (42.9%)	11 (40.7%)	
Crutch	33 (53.2%)	19 (54.3%)	14 (51.9%)	
Walker	3 (4.8%)	1 (2.9%)	2 (7.4%)	
Sedentary Behaviour (Yes), *n* (%)	250 (52.7%)	88 (50.3%)	162 (54.2%)	0.412
Upper extremities assistance to stand up from a chair (Yes), *n* (%)	205 (43.2%)	75 (42.9%)	130 (43.5%)	0.895
BMI, mean (SD) (*n* = 458)	27.82 (4.21)	28.32 (4.31)	27.51 (4.12)	0.044
Alcohol consumption (Yes), *n* (%)	42 (8.9%)	4 (2.3%)	38 (12.7%)	<0.001

Abbreviations: BMI: Body Mass Index (kg/m^2^).; SD: standard deviation.

**Table 2:** Fall history, overall and stratified by living environment (rural vs. urban).

Characteristics	Overall (*n* = 474)	Rural (*n* = 175)	Urban (*n* = 299)	*p*
History of falls in the last 12 months (Yes), *n* (%)	185 (39.0%)	64 (36.6%)	121 (40.5%)	0.401
Number of falls, mean (SD) (*n* = 183)	2.24 (2.41)	1.84 (1.32)	2.45 (2.81)	0.048
Fear of falls (Yes), *n* (%)	261 (55.1%)	106 (60.6%)	155 (51.8%)	0.065
Where falls occurred, *n* (%):				0.314
Inside	64 (34.8%)	25 (39.7%)	39 (32.2%)	
Outside	120 (65.8%)	38 (60.3%)	82 (67.8%)	
Reason for fall, *n* (%) (*n* = 184):				0.603
Slip	44 (23.9%)	17 (26.6%)	27 (22.5%)	
Stumble	78 (42.4%)	28 (43.8%)	50 (41.7%)	
Loss of consciousness	3 (1.6%)	0 (0%)	3 (2.5%)	
Dizziness	12 (6.5%)	3 (4.7%)	9 (7.5%)	
Lower extremity weakness	13 (7.1%)	4 (6.3%)	9 (7.5%)	
No special reason	20 (10.9%)	9 (14.1%)	11 (9.2%)	
Other	14 (7.6%)	3 (4.7%)	11 (9.2%)	
Need for health services assistance (Yes), *n* (%) (*n* = 185)	63 (34.1%)	23 (35.9%)	40 (33.1%)	0.694
Which health service, *n* (%) (*n* = 63):				0.396
Hospital Emergency	55 (87.3%)	19 (82.6%)	36 (90.0%)	
Primary healthcare centre	8 (12.7%)	4 (17.4%)	4 (10.0%)	
Hospitalization (Yes), *n* (%) (*n* = 56)	10 (17.9%)	2 (10.5%)	8 (21.6%)	0.305
Activities limited & restricted due to fall (Yes), *n* (%) (*n* = 184)	43 (23.4%)	14 (21.9%)	29 (24.2%)	0.726

Abbreviations: SD: standard deviation.

**Table 3:** Social participation, functional capacity and self-efficacy for exercise, overall and stratified by living environment (rural vs. urban).

Characteristics	Overall (*n* = 474)	Rural (*n* = 175)	Urban (*n* = 299)	*p*
PAPM, *n* (%) (*n* = 470):				0.013
no restrictions	239 (50.9%)	80 (46.8%)	159 (53.2%)	
mild restrictions	136 (28.9%)	43 (25.1%)	93 (31.1%)	
moderate restrictions	64 (13.6%)	31 (18.1%)	33 (11.0%)	
severe restrictions	31 (6.6%)	17 (9.9%)	14 (4.7%)	
TUG, mean (SD) (*n* = 457)	10.99 (7.40)	13.4 (10.40)	9.62 (4.43)	<0.001
Grip strength, mean (SD)	22.74 (8.27)	21.03 (7.36)	23.73 (8.61)	<0.001
Walking speed, mean (SD)	1.35 (0.43)	1.17 (0.44)	1.45 (0.39)	<0.001
Sit to stand, mean (SD) (*n* = 450)	10.94 (3.96)	9.25 (3.47)	11.87 (3.90)	<0.001
Self-efficacy for exercise, mean (SD) (*n* = 471)	13.75 (4.66)	12.83 (4.97)	14.28 (4.40)	0.002

Abbreviations: SD: standard deviation; TUG: Timed Up and Go test; PAPM: Activities and Participation Profile related to Mobility.

## Data Availability

The original contributions presented in this study are included in the article. Further inquiries can be directed to the corresponding author.

## Appendix A. Description of Functional Tests Selected from the FallSensing Protocol

* The sample comprised 537 participants; the sample size was determined for a finite population with a 95% confidence interval and a 5% margin of error (Source: INE, PORDATA). To be representative, the minimum required sample size was 385; however, our study included 537 participants.

**Handgrip Strength (HS):** Participants performed the handgrip strength test while seated in a standard armless chair, with shoulders adducted and neutrally rotated, elbows flexed at 90°, and wrists positioned in 0–15° of ulnar deviation. A hand dynamometer was used, set to the second handle position and held in the dominant hand in a vertical orientation. Participants were instructed to exert maximal grip strength for 5 s. The final result was recorded in kilograms of force (kgf). Normative reference values differ by sex; values below 15 kg in women and below 21 kg in men indicate an increased risk of falls [52]. In the Portuguese population, the mean grip strength was 24.2 kg (SD = 8.82).

**10-Meter Walk Speed (10-mWS) Test:** Gait speed was measured along a 20-m walkway, with the initial and final 5 m designated for acceleration and deceleration. Timing was recorded between the 5- and 15-m marks. Participants wore comfortable footwear and were allowed to use assistive devices. Walking speed ≤ 1 m/s indicates higher fall risk, whereas speeds ≥ 1.42 m/s are considered sufficient for safe street crossing [53]. In the Portuguese population, the mean walking speed was 1.44 m/s (SD = 0.43).

**30-Second Sit-to-Stand (30 s STS) Test:** The 30-s Sit-to-Stand (30 s STS) Test evaluates lower limb strength by counting the number of times participants can rise from a seated to a standing position and return to sitting within 30 s. The final score corresponds to the total number of complete repetitions performed. Normative values differ according to age and sex [54]. In the Portuguese population, the mean number of repetitions in the 30 s STS test was 11.6 (SD = 4.09).

**Timed Up and Go TUG:** The TUG test measures dynamic balance, mobility, and lower limb strength. Participants started seated in a standard chair and were instructed to stand up, walk 3 m, turn around, return to the chair, and sit down as quickly and safely as possible, without running or using upper limb support. A completion time greater than 12 s indicates an increased risk of falls in older adults [55]. In the Portuguese population, the mean time to complete the Timed Up and Go test was 9.84 s (SD = 6.43).

## Appendix B. Analyses of Covariance (ANCOVA) to Examine the Effects of Age and Place of Residence (Rural vs. Urban) on Health Conditions, Lifestyle Behaviours, Mobility Aids, Social Participation, Functional Capacity and Self-Efficacy for Exercise Outcomes

Significant effects of both age and residence were observed for Timed Up and Go (TUG) (Age: F(1,454) = 65.40, *p* < 0.001; Residence: F(1,454) = 12.53, *p* < 0.001), Grip Strength (Age: F(1,471) = 25.70, *p* < 0.001; Residence: F(1,471) = 4.35, *p* = 0.037), Sit-to-Stand (Age: F(1,447) = 28.99, *p* < 0.001; Residence: F(1,447) = 33.98, *p* < 0.001), and Walking Speed (Age: F(1,471) = 81.57, *p* < 0.001; Residence: F(1,471) = 24.59, *p* < 0.001), indicating that older participants performed worse across these measures, and urban versus rural differences remained significant even after controlling for age.

Age significantly influenced PAPM (F(1,467) = 24.77, *p* < 0.001) and the use of mobility assistive devices (F(1,471) = 50.39, *p* < 0.001), with older participants demonstrating higher scores and greater device use. Conversely, age did not significantly affect Self-Efficacy for Exercise (F(1,468) = 2.78, *p* = 0.096), BMI (F(1,455) = 1.09, *p* = 0.297), hearing problems (F(1,471) = 1.28, *p* = 0.259), or hypertension (F(1,471) = 1.35, *p* = 0.245).

Regarding the effect of residence, significant differences between rural and urban participants were found for Self-Efficacy (F(1,468) = 7.61, *p* = 0.006), BMI (F(1,455) = 4.91, *p* = 0.027), the use of ≥4 medications (F(1,471) = 5.12, *p* = 0.024), and hearing problems (F(1,471) = 12.30, *p* < 0.001). No significant effects of residence were observed for PAPM_total (F(1,467) = 1.40, *p* = 0.237), mobility assistive device use (F(1,471) = 2.20, *p* = 0.139), poor vision (F(1,471) = 3.68, *p* = 0.056), or education (F(1,471) = 1.39, *p* = 0.240).

In summary, some outcomes were primarily influenced by age, including TUG, Grip Strength, Sit-to-Stand, Walking Speed, PAPM, and mobility device use. Other outcomes were primarily influenced by residence, such as Self-Efficacy, BMI, use of ≥4 medications, hearing problems, and hypertension. Several outcomes were affected by both age and residence (e.g., physical function measures and use of multiple medications), while some were influenced predominantly by age or residence alone. These results indicate that age and rural-urban context differentially contribute to functional, health, and psychosocial outcomes in the studied population.
